# Effects of multi-organ crosstalk on the physiology and pathology of adipose tissue

**DOI:** 10.3389/fendo.2023.1198984

**Published:** 2023-06-13

**Authors:** Sufen Wang, Yifan Liu, Jiaqi Chen, Yuejing He, Wanrui Ma, Xinguang Liu, Xuerong Sun

**Affiliations:** ^1^ Guangdong Provincial Key Laboratory of Medical Molecular Diagnostics, The First Dongguan Affiliated Hospital, Guangdong Medical University, Dongguan, China; ^2^ Institute of Aging Research, School of Medical Technology, Guangdong Medical University, Dongguan, China; ^3^ Clinical Laboratory, Dongguan Eighth People’s Hospital, Dongguan, China; ^4^ Department of General Medicine, The First Dongguan Affiliated Hospital, Guangdong Medical University, Dongguan, China

**Keywords:** adipose tissue, organs, crosstalk, adipocyte, obesity, diabetes

## Abstract

In previous studies, adipocytes were found to play an important role in regulating whole-body nutrition and energy balance, and are also important in energy metabolism, hormone secretion, and immune regulation. Different adipocytes have different contributions to the body, with white adipocytes primarily storing energy and brown adipocytes producing heat. Recently discovered beige adipocytes, which have characteristics in between white and brown adipocytes, also have the potential to produce heat. Adipocytes interact with other cells in the microenvironment to promote blood vessel growth and immune and neural network interactions. Adipose tissue plays an important role in obesity, metabolic syndrome, and type 2 diabetes. Dysfunction in adipose tissue endocrine and immune regulation can cause and promote the occurrence and development of related diseases. Adipose tissue can also secrete multiple cytokines, which can interact with organs; however, previous studies have not comprehensively summarized the interaction between adipose tissue and other organs. This article reviews the effect of multi-organ crosstalk on the physiology and pathology of adipose tissue, including interactions between the central nervous system, heart, liver, skeletal muscle, and intestines, as well as the mechanisms of adipose tissue in the development of various diseases and its role in disease treatment. It emphasizes the importance of a deeper understanding of these mechanisms for the prevention and treatment of related diseases. Determining these mechanisms has enormous potential for identifying new targets for treating diabetes, metabolic disorders, and cardiovascular diseases.

## 1 Introduction

As critical energy reservoir, adipose tissue plays a crucial role in maintaining energy homeostasis and metabolism in the human body. White adipose tissue is a major site of energy storage and stores large numbers of white adipocytes (1). Brown adipocytes are present in brown adipose tissue and are the main thermogenic adipocytes, mainly found in specific areas under the skin of newborns and in smaller numbers in adults (2). Beige adipocytes are a metabolic state between white and brown adipocytes, which do not form a distinct fat depot, but appear within white adipose tissue and differentiate from white adipocytes to beige adipocytes in response to cold or β3-adrenergic stimulation, called “browning” (3). Adipocyte thermogenesis is mainly associated with the expression of uncoupling protein 1 (UCP1) in the mitochondria of brown adipocytes and beige adipocytes. It has been shown that brown or beige adipocytes activity is associated with the prevention of obesity and the development of metabolic diseases.

**Figure 1:**
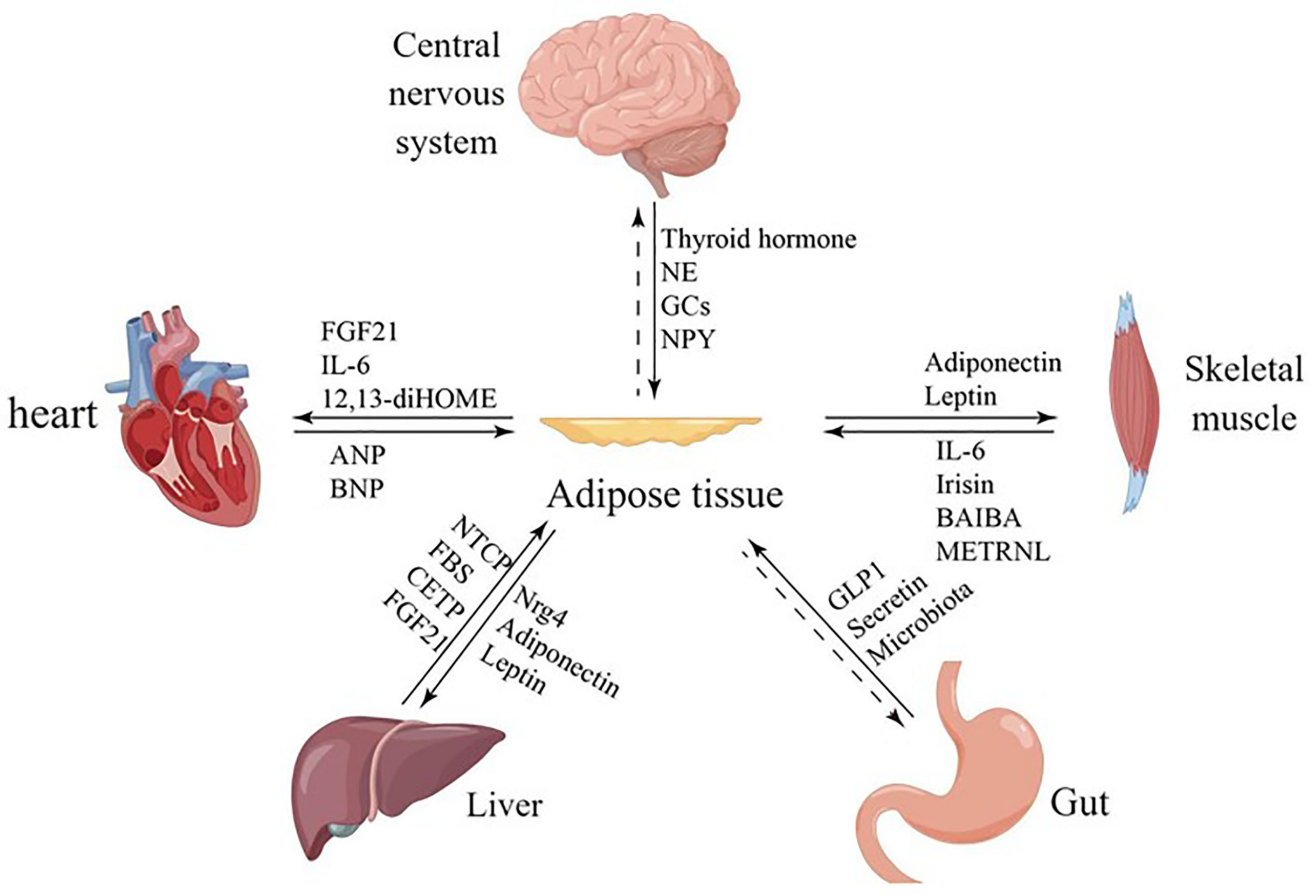
Communication between adipose tissue and other organs. Adipose tissue and other organs crosstalk by secreting related factors.

Furthermore, adipocytes participates in various physiological processes, including growth and development, immune regulation, and inflammation, and influences the function of other organs by secreting hormones, cytokines, and fatty acids (4–6) (**Figure 1**). White adipocytes secretes adipokines, such as leptin and lipocalin, which are released in response to a certain energy state. These adipokines play a crucial endocrine function, regulating the central nervous system or the metabolic activity of peripheral organs to maintain energy balance (7) (**Table 1**). Brown adipose tissue may also have secretory effects, such as irisin, fibroblast growth factor 21, IL-6, and neuregulin 4, which interact with other organs (10) (**Table 1**). Adipose tissue can also release different endocrine signaling molecules, including proteins, lipids and microRNAs, into the circulation to exert regulatory effects on target tissues or organs (1). Similarly, other organs secrete corresponding cytokines to regulate adipose tissue thermogenesis (**Table 1**).

**Table 1:** Metabolic functions of adipokines and cytokines and their crosstalk with other organs.

Adipokines	Metabolic functions	Affected tissue	References
FGF21	Improve heart function,Inducing an increase in mitochondrial cristae to increase	heart,Muscle	(8, 9)
IL-6	Increased cardiac matrix oxidation, protecting the heart from hypertrophy and oxidative stress	herat	(10)
12, 13-diHOME	Increased cardiac hemodynamics,Increased skeletal muscle fatty acid uptake and oxidation	heart,Muscle	(11, 12)
Leptin	Protecting the heart from inflammation, Prevent excessive accumulation of lipids in the liver,Promotes skeletal muscle lipid metabolism and fatty acid utilization	heart,live,Muscle	(13–16)
NRG4	Reduces insulin resistance and liver steatosis	liver	(17)
Lipocalin	Inhibits liver cell damage and death,Reduces ceramide levels in exercise-oxidized muscles	liver,Muscle	(14, 18–20)
RBP4	Regulation of glucose homeostasis and insulin uptake	Muscle	(21)
Endolipin	Regulation of glucose homeostasis and insulin uptake	Muscle	(21)
IRF4	Maintenance of skeletal muscle motility	Muscle	(22)
TNF-α	induce insulin resistance and lead to metabolic disorders	WAT,BAT	(23)
resistin	induce insulin resistance and lead to metabolic disorders	WAT,BAT	(23)
Cytokines			
E2	Regulating energy balanceand regulating thermogenesis in brown adipose tissue (BAT)	BAT	(24)
ANP	Inducing mitochondrial biogenesis, and increasing uncoupling and total respiration	BAT,WAT	(25)
BNP	Inducing mitochondrial biogenesis, and increasing uncoupling and total respiration	BAT,WAT	(25)
Bile acids	Induce energy expenditure by promoting intracellular thyroid hormone activation and promoting mitochondrial fission and beige remodeling of white adipose tissue	BAT,WAT	(26, 27)
CETP	Enhancing fat breakdown and activating brown adipose tissue to alleviate obesity	BAT	(28)
IL-6	Regulating brown adipose tissue metabolism and increasing the expression of UCP1	WAT	(29, 30)
Irisin	Inducing white adipocytes to undergo browning	WAT	(31)
BAIBA	Promotion of brown adipose tissue activation, white adipose tissue browning, and induction of lipolysis.	WAT,BAT	(32)
METRNL	Promote selective activation of adipose tissue macrophages, leading to increased expression of thermogenic and anti-inflammatory genes.	WAT,BAT	(33)
BDNF	Induce white adipose tissue browning.	WAT	(34)
IL-15	Enhance lipid breakdown and inhibit lipid synthesis.	WAT,BAT	(35)
SPARC	Negative regulation of adipocyte differentiation and fat synthesis in white adipose tissue (WAT).	WAT	(36)
GLP-1	Regulate thermogenesis in white and brown adipose tissue.	WAT,BAT	(37, 38)

However, abnormal adipose tissue function can cause several health problems. Many diseases, such as type 2 diabetes, hypertension, and atherosclerosis, are closely related to abnormal adipose tissue function. During obesity, adipose tissue secretes a large amount of proinflammatory adipokines such as tumor necrosis factor-α (TNF-α) and resistin, which induce insulin resistance and lead to metabolic disorders (23). Therefore, in-depth research into the mechanisms of adipose tissue function and interorgan interactions in both physiological and pathological states is of great significance for improving human health. This review discusses the physiological and pathological characteristics of adipose tissue, the ecological niche of adipose tissue, the interaction between adipose tissue and multiple organs, as well as the role of adipose tissue in the development and treatment of diseases from four aspects. Firstly, it introduces the characteristics of adipose tissue as a critical energy reservoir and the different types of adipocytes, and discusses the importance of adipose tissue in overall energy metabolism. Secondly, it analyzes the importance of adipose tissue in interorgan interactions and the effects of different types of adipocytes on metabolism and disease, and explores the underlying molecular mechanisms. Finally, based on current research, we discuss the potential of transplanting brown adipocytes or beige adipocytes as a novel therapy for obesity and metabolic diseases.

## 2 Physiological characteristics of adipocytes

### 2.1 White adipocytes

Adipose tissue is divided into white adipose tissue (WAT) and brown adipose tissue (BAT). White adipose tissue plays a key role in the energy status and metabolism of the whole body; it not only stores excess energy, but also secretes various hormones and metabolites to regulate the energy balance of the body (39). White adipose tissue is composed of large adipocytes with single lipid droplets and fewer mitochondria, which endow it with the ability to store energy and maintain homeostasis in response to nutritional requirements (40). In humans, white adipose tissue can be classified according to its distribution in two storage reservoirs: visceral white adipose tissue (VAT), which includes omental, mesenteric, retroperitoneal, gonadal and pericardial white adipose tissue, and subcutaneous white adipose tissue (SAT), which is located under the skin (41).

### 2.2 Brown adipocytes

Brown adipose tissue dissipates energy by producing heat, which is known as non-shivering thermogenesis, characterized by multilocular lipid droplets and abundant mitochondria (42). This thermogenesis occurs through the expression of uncoupling protein 1 (UCP1), a protein found in the inner mitochondrial membrane, which dissipates the proton electrochemical gradient generated by respiration in the form of heat (43). Brown adipose tissue has a large number of mitochondria and more capillaries than white adipose tissue due to a greater demand for oxygen. In addition, brown adipose tissue has a denser nerve supply than white adipocytes. The brown color of brown adipocytes is attributed to their high mitochondrial density and high vascularization (44). Brown adipose tissue is abundant in small mammals or neonates that are prone to decreased body temperature due to larger body surface volume ratios and have difficulty maintaining adequate core body temperature through white adipose tissue isolation or muscle shivering. In human fetuses and neonates, brown adipose tissue is present in the axillae, neck, perinephric and periadrenal regions, but decreases shortly after birth (43). In adult humans, brown fat is found in the neck, supraclavicular, axillary, paravertebral, mediastinal, and epigastric regions (45). And it has been shown that the amount of brown adipose tissue decreases and weight increases with age, but the causal relationship has not been concluded (42).

### 2.3 Conversion of white adipocytes to brown adipocytes

Beige adipocytes are induced thermogenic adipocytes found sporadically in white adipose tissue, containing multi-compartment lipid droplets and dense mitochondria expressing uncoupling protein-1. The development of beige adipocytes is called “browning”. Beige adipocytes are induced by environmental stimuli such as chronic colds and β3-adrenoceptor agonists (46). Activated beige adipocytes burn free fatty acids to generate heat, but the thermogenic capacity of beige adipocytes is only one-fifth that of brown adipocytes (47). Moreover, compared with brown adipocytes, the maintenance of beige adipocyte morphology is transient, which may be due to mitochondrial biogenesis being active in brown adipocytes while mitochondrial clearance is predominant in maintaining beige adipocytes. After removing environmental stimuli, beige adipocytes will be converted into white adipocytes again, which is closely related to the significant decrease in mitochondrial content and increase in mitochondrial autophagy. The adipocyte-specific loss of Atg5 or Atg12, which blocks the autophagy pathway that expresses UCP-1, can prevent beige adipocytes from being converted into white adipocytes (48).

However, it is still debated whether beige cells arise through redifferentiation from adipose precursor cells or through transformation between mature white adipocytes. Browning involves the expression of many transcription factors, such as the PR structural domain containing 16 (PRDM16)^1^ and the peroxisome proliferator-activated receptor PPAR-γ, as well as the expression of uncoupling protein 1 (UCP1), a marker of thermogenesis (49). Characteristics of white adipose tissue browning include the presence of smaller multilocular adipocytes, increased UCP1 mRNA expression and mitochondrial density, and enhanced respiratory capacity (50). It has been shown that microbiota depletion promotes white adipose tissue browning and reduces obesity. This resulted in increased glucose tolerance, insulin sensitivity and reduced white adipose tissue in normal mice, obese and high-fat diet-fed mice (51). The thermogenic response of brown adipocytes to thyroid hormone is the result of a synergistic effect of thyroid hormone and the sympathetic nervous system. In recent years, it has been shown that thyroid hormones also cause browning of white adipose tissue (52). There is increasing evidence that thermogenic adipocytes in the human white adipose tissue depot can be induced under specific stimuli and that chronic cold exposure is one of the most effective physiological inducers of adipose tissue browning. These observations raise important questions about the great plasticity of human adipose depots and about the metabolic properties of thermogenic adipose tissue in humans and their potential therapeutic implications (53).

### 2.4 Role of adipose tissue in physiology and pathology

Chronic low-grade inflammation of adipose tissue is one of the mechanisms that lead to the progression of metabolic diseases such as type II diabetes and cardiovascular disease (54). Obesity and aging trigger adipose tissue alterations that eventually lead to a pro-inflammatory phenotype of adipose tissue immune cells. Disturbed adipose tissue metabolism, increased ratio of visceral adipose tissue to subcutaneous adipose tissue, and shortened lifespan are all characteristics of obesity and aging (55). In obese patients, the homeostatic mechanisms between adipose tissue function and secretome and the control of local cell-cell interactions are dysregulated. Systemic or local inflammation and insulin resistance lead to a shift from anti-inflammatory and anti-atherogenic to pro-inflammatory and pro-atherogenic adipose tissue secretory bodies (56).

White adipose tissue is the major fat storage reservoir and the largest endocrine organ for adipokines and cytokines. Adipokines are involved in various metabolic and physiological signaling cascades and regulate insulin signaling, glucose uptake, fatty acid oxidation, and other energy production and metabolic processes. Obesity results in a white adipose tissue phenotypic transition characterized by the presence of inflammation, dysfunctional adipocytes, and immune cell infiltration in the vascular portion of the stroma. Adipocytes secrete pro-inflammatory cytokines locally and systemically, which in turn disrupt the normal function of the adipose tissue itself and distal organs (57).

Obesity can lead to adipocytes hypertrophy and hyperplasia (58). Hyperplasia results in the redifferentiation of adipocytes from progenitor cells, while hypertrophy refers to the increase in size of each existing adipocyte to accommodate the increasing amount of fuel. Proliferative adipose tissue expansion occurs primarily during development and hypertrophic expansion occurs primarily after development and depends on the ability of existing adipocytes to capture and retain circulating lipids (59, 60). The expansion of adipose tissue is a mechanism designed to buffer nutrient excess. When there is an imbalance between caloric intake and energy expenditure, obesity can occur, which further promotes the expansion of adipose tissue. Adipose tissue expansion occurs primarily in subcutaneous and visceral fat. Studies have shown that adipose tissue distribution is a strong predictor of the development of metabolic syndrome. In obese patients, visceral adipose tissue expansion is associated with an increased risk of developing cardiovascular and metabolic diseases and their serious complications, whereas obese patients with predominantly subcutaneous fat stores may have a reduced risk of metabolic disease or delayed complications (7).

## 3 Effects of intercellular crosstalk in the adipose ecotone on its physiology and pathology

### 3.1 Adipocyte ecotone

Adipose tissue ecotone refers to the microenvironment of adipocytes with other cells in adipose tissue (1). Adipose tissue is composed not only of adipocytes but also of immune cell cells, endothelial cells, smooth muscle cells, and adipose progenitor cells. These cell populations produce dynamic changes during the aging process or in the development of obesity. Adipocytes play a major role in maintaining energy homeostasis and also form an adipocyte ecotone with other cell types and regulate adipose tissue function through extensive cellular crosstalk. This is important for regulating the expansion and remodeling of adipose tissue as well as in response to external stimuli such as ambient temperature and diet. Therefore, the study of cell populations in adipose tissue and intercellular crosstalk in the tissue ecotone is important for understanding the regulatory state of the organ (61).

### 3.2 Crosstalk between adipocytes and sympathetic nerves

#### 3.2.1 Sympathetic nerves release multiple factors to regulate adipocyte function

Studies have shown that sensory nerves and sympathetic nerve fibers play a dominant role in regulating adipose function. Sympathetic nerve endings regulate adipocyte function through the release of factors such as norepinephrine, neuropeptide Y (NPY) and ATP (62), β-adrenergic receptors (βAR) are channels that mobilize fat for consumption in other tissues, and their activation promotes lipolysis of stored triglycerides in white and brown adipocytes (63). Norepinephrine-mediated lipolysis in white adipose tissue is dependent on β-adrenoceptor stimulation and ultimately on hormone-sensitive lipase and phospholipid A phosphorylation. And in addition to controlling lipolysis, the increase or decrease of norepinephrine inhibits and stimulates white adipocyte proliferation, respectively (64). In contrast to norepinephrine, NPY promotes adipocyte differentiation and lipid accumulation, leading to energy storage in adipose tissue, whose effects are mediated mainly through NPY receptor subtypes 1 and 2 (65).

#### 3.2.2 Sympathetic nerves mediates the production of bioactive lipids by adipocytes to exert its effects

The endocannabinoid system (ECS) plays a key role in the development of obesity, and blocking type 1 cannabinoid receptors can reduce body weight and attenuate metabolic disorders associated with obesity. Adipocyte production of endocannabinoids negatively regulates adiposympathetic innervation, thereby inhibiting brown adipose tissue thermogenesis and promoting white adipose tissue accumulation (66). In addition, adipocyte-secreted BMP8b^2^ mediates adrenergic-induced remodeling of neurovascular networks in adipose tissue. The innervation and vascular remodeling effects require BMP8b signaling through adipocytes to secrete neuromodulatory protein-4 (NRG4)^3^, which promotes sympathetic axon growth and branch formation *in vitro (* 67). In addition, adipocytes also secrete various neurotrophic factors such as nerve growth factor and S100 calcium-binding protein b (S100b) to promote synapse growth and S100b to innervate thermogenic adipose tissue (68–70).

### 3.3 Crosstalk between adipocytes and vascular cells

#### 3.3.1 Adipocytes regulate vascular cell function by secreting cytokines

Adipocytes produce a large number of pro-angiogenic factors, including fibroblast growth factor 2 (FGF-2), VEGF, hepatocyte growth factor (HGF) and PDGF (71). Studies have shown that exposure of mice to cold leads to activation of angiogenesis in white and brown adipose tissue, with upregulation of pro-angiogenic factors such as VEGF and downregulation of endogenous angiogenic inhibitors including thrombomodulin. reduction of VEGFR2 abolished cold-induced angiogenesis and significantly impaired non-shivering thermogenesis. reduction of VEGFR1 led to the opposite effect: increased adipose tissue vascularity and non-shivering thermogenic capacity was increased (72). VEGFA in brown adipocytes is a highly specific and potent angiogenic factor that promotes the specificity of vascular endothelial cell proliferation, migration and survival, VEGFA overexpression enhances mitochondrial respiration and thermogenesis, VEGFB plays an important role in adipose tissue vascular remodeling and specifically controls endothelial uptake of fatty acids through transcriptional regulation of vascular fatty acid transport proteins (73).

#### 3.3.2 Endothelial cells regulate adipocyte function by secreting endothelin-1 and nitric oxide

Endothelin-1 (ET-1) and epidermal growth factor (EGF) secreted by vascular endothelial cells have important regulatory effects on adipocytes. They block the expression of C/EBPα and the adipose marker PPARγ, there by inhibiting adipogenesis. Additionally, Endothelin-1 inhibits the differentiation of human and mouse adipocyte progenitor cells (74). Endothelin-1 has a direct effect on adipocytes, and long-term treatment of adipocytes with endothelin-1 *in vitro* leads to desensitization of insulin signaling, resulting in reduced glucose transport. Endothelin-1 stimulates lipolysis by binding to endothelin receptor A (75). Plasma endothelin-1 levels are elevated in patients with obesity and type 2 diabetes, but the primary source of circulating endothelin-1 in these conditions is unknown (76). Studies have shown that nitric oxide secreted by vascular endothelial cells can induce vascular smooth muscle relaxation through guanylate cyclase activation and cyclic GMP formation, thereby promoting vasodilation and enhancing thermogenesis (77). Nitric oxide can regulate the number and function of mitochondria in brown adipocytes and various cell lines. Mitochondria play a key role in brown adipose thermogenesis, so nitric oxide can affect adipose thermogenesis (78).

### 3.4 Crosstalk between adipocytes and immune cells

#### 3.4.1 M1 macrophages secrete pro-inflammatory cytokines to inhibit adipocyte thermogenesis

Local infiltration of immune cells and production of pro-inflammatory cytokines can contribute to obesity and insulin resistance, with macrophages being most closely associated with obesity and insulin resistance (79). Macrophages can exhibit both M1 and M2 phenotypes, and the transition from an anti-inflammatory M2-like phenotype to a pro-inflammatory M1-like phenotype is believed to be the primary cause of adipose tissue inflammation in obese patients. In addition, the number of macrophages is positively correlated with insulin resistance (80). Local infiltration of immune cells and increased production of pro-inflammatory cytokines contribute to the development of insulin resistance in obesity. Compared to white adipose tissue, brown adipose tissue is less susceptible to local inflammation caused by obesity. However, the production of pro-inflammatory cytokines can ultimately lead to a pro-inflammatory environment in brown fat, which can directly impact its thermogenic activity by impairing energy expenditure mechanisms and disrupting glucose metabolism. This is thought to contribute to the development of obesity-related metabolic disorders (81). Interleukin-1 beta (IL-1β) is a typical pro-inflammatory cytokine released by M1 macrophages. Its mRNA expression levels are upregulated in the white adipose tissue of obese mice and RAW264.7 macrophages. IL-1β can inhibit the expression of uncoupling protein 1 (UCP1) induced by beta-adrenergic receptor stimulation in adipocytes, which impairs thermogenic function both *in vivo* and *in vitro (* 82).

#### 3.4.2 Enhanced BAT activation and WAT browning by M2 macrophages

The browning process of beige adipocytes is associated with an increase in anti-inflammatory M2 macrophages, but the function of M2 macrophages in the browning process and their underlying mechanisms are not fully understood. It has been shown that the macrophage cytokine Slit3 binds to ROBO1 receptors on sympathetic neurons and activates tyrosine hydrolase (TH) *via* PKA and CaMKII signaling, thereby stimulating the synthesis and release of norepinephrine, which acts on adipocytes and stimulates thermogenesis (83). Mechanistically, macrophages in white adipose tissue undergo alternative activation under cold stress, which induces the expression of tyrosine hydroxylase and the production of catecholamines. These catecholamines are required for the process of white adipose tissue browning (84). In one study, tyrosine hydroxylase expression was not detected in macrophage populations isolated from brown adipose tissue and white adipose tissue after cold exposure. This suggests that alternatively activated M2 macrophages may be the source of catecholamines in white adipose tissue (85).

CXCL14 (C-X-C motif chemokine ligand 14)^4^, also known as Breast and Kidney-expressed chemokine (BRAK), was identified in a 2018 study as a novel regulatory factor secreted during the thermogenic activation of brown fat cells. CXCL14 released by brown fat cells recruits alternatively activated M2 macrophages and promotes browning of white fat in high-fat diet-induced obese mice, leading to improved glucose and insulin homeostasis (86). It has also been shown that lipocalin enhances cold-induced subcutaneous adipose tissue browning by promoting M2 macrophage proliferation (87).

## 4 Effects of crosstalk between adipose tissue and organs on their physiology and pathology

### 4.1 Crosstalk between adipose tissue and brain

#### 4.1.1 Regulation of adipocyte energy metabolism and thermogenesis by the central nervous system

**Figure 2:**
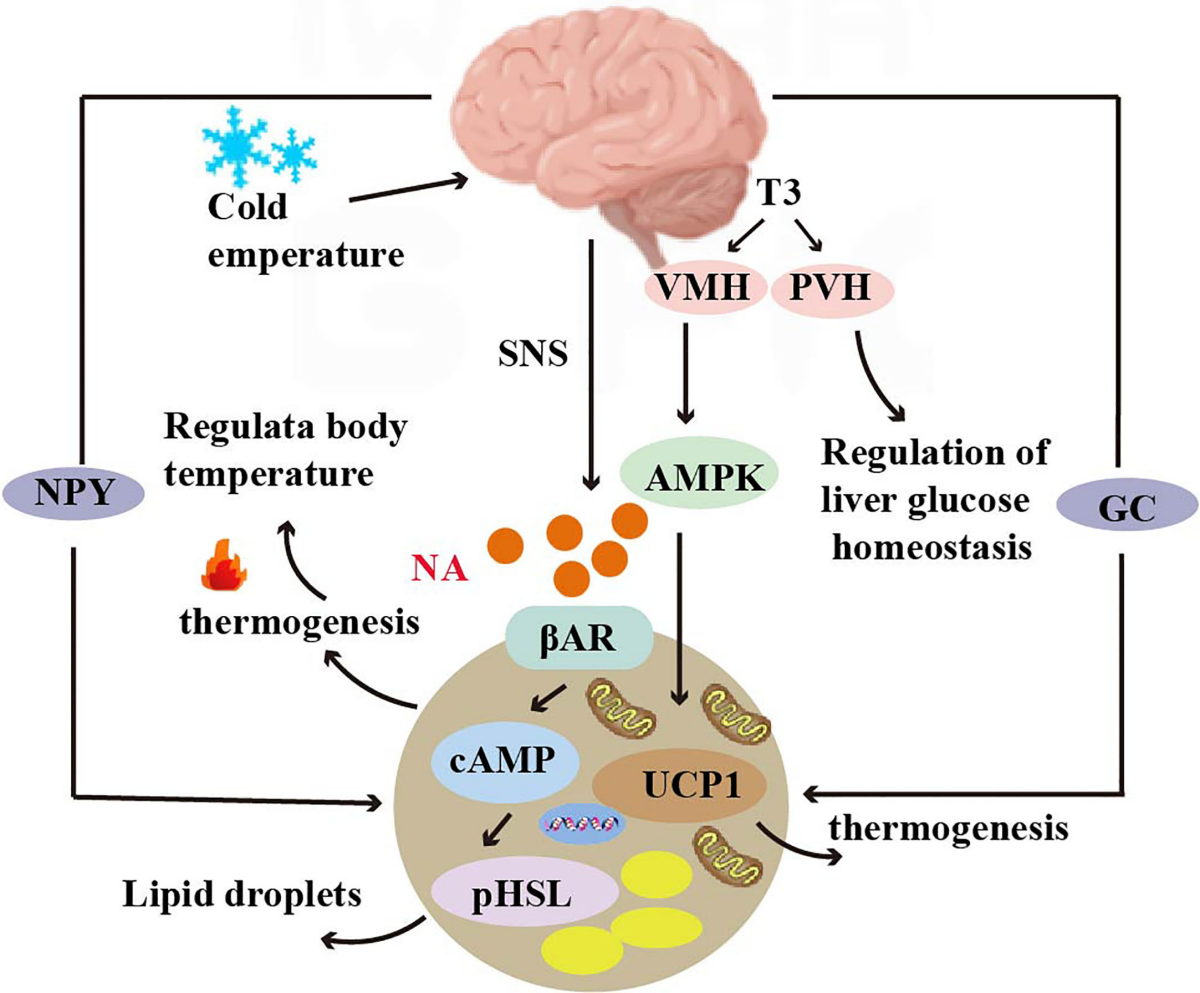
Crosstalk between adipose tissue and brain. Norepinephrine can bind to β3 adrenergic receptors (β3 AR) and increase cAMP levels, activating protein kinase A (PKA) and hormone-sensitive lipase (HSL) to mediate lipolysis. T3 regulates thermogenic programs in brown adipose tissue (BAT) through AMP-activated protein kinase (AMPK) in the ventromedial hypothalamus (VMH), and T3 in the paraventricular nucleus (PVH) of the hypothalamus regulates glucose homeostasis in the liver. T3 induces the expression of the UCP1 gene and regulates thermogenesis. Glucocorticoids and neuropeptide Y (NPY) mediate thermogenesis and regulate the function of brown adipose tissue (BAT).

The central nervous system is able to integrate feeding information to influence feeding behavior and long-term energy homeostasis (88). The hypothalamus is involved in thermoregulation by activating brown adipose tissue in response to peripheral cold-sensing signals, resulting in non- shivering thermogenesis (89). The hypothalamus also contributes to the maintenance of body temperature by driving sympathetic activation that allows the release of norepinephrine from nerve terminals innervating the BAT, which activates the intracellular lipolytic cascade and releases fatty acids that fuel the brown fat mitochondrial electron transport chain (90) (**Figure 2**).

Thermogenesis of BAT is activated by the hypothalamus *via* the sympathetic nervous system. Norepinephrine, a key neurotransmitter mediating activation, binds to the b3-adrenoceptor (b3 AR) to increase cAMP levels, thereby rapidly activating protein kinase A (PKA) and hormone-sensitive lipase (HSL)-mediated lipolysis. It has been shown that CNS activation for BAT thermogenesis is mainly associated with the thyroid hormone T3, which regulates the thermogenic program in brown adipose tissue (BAT) *via* AMP-activated protein kinase (AMPK) in the ventral medial nucleus of the hypothalamus (VMH). In addition, T3 acting in the paraventricular nucleus of the hypothalamus (PVH) also regulates glucose homeostasis in the liver (91). T3 also regulates thermogenesis by inducing the expression of the UCP1 gene (92) (**Figure 2**).

#### 4.1.2 Hormone-mediated central effects contribute to thermogenesis

Estrogen-mediated central actions also play an important role for BAT, and it has been shown that estradiol (E2) can regulate energy homeostasis by reducing hypothalamic ceramide levels and endoplasmic reticulum (ER) stress. Most of the effects of estradiol on feeding occur in pre-opioid melanopsin (POMC) neurons in the hypothalamic arcuate nucleus, but regulation of thermogenesis in BAT occurs in the ventral medial nucleus of the hypothalamus (24). A 2015 study showed that insulin and leptin^5^ secreted by adipocytes acting together on hypothalamic neurons can promote WAT browning and weight loss. The mechanism of action is that leptin acts on the POMC to suppress food intake and increase energy expenditure by promoting thermogenesis in the BAT, and leptin action in the hypothalamus increases sympathetic activity in the BAT, as well as increases UCP-1 expression. Insulin acts on the POMC to regulate systemic glucose metabolism and trigger an anorexic response (93).

Glucocorticoids are a class of steroidal compounds synthesized and secreted by the fasciculata zone of the adrenal cortex. *In vivo*, the hypothalamic-anterior pituitary-adrenocortical axis primarily regulates glucocorticoid secretion. It was once demonstrated that in rodents, glucocorticoids inhibit UCP1 expression in brown adipocytes; however, this demonstration was overturned in a 2019 study (94). Glucocorticoids have opposite effects in humans and rodents, and the glucocorticoid prednisolone sharply increased fluorodeoxyglucose uptake *via* BAT in healthy men under mild cold exposure. In addition, prednisolone increased human supraclavicular skin temperature and energy expenditure during cold exposure. And glucocorticoids increased isoprenaline-stimulated respiration and UCP-1 in human primary brown adipocytes, but significantly decreased isoprenaline-stimulated respiration and UCP-1 in murine primary brown and beige adipocytes (95). A study using positron emission tomography/computed tomography (PET/CT) to noninvasively study BAT activity and brain glucose metabolism *in vivo* found that neuropeptide Y (NPY) secreted by the hypothalamus mediates brain glucose metabolism consistent with BAT activity in healthy adults (96) (**Figure 2**).

### 4.2 Crosstalk between adipose tissue and heart

#### 4.2.1 Effect of adipose tissue on cardiac function

**Figure 3:**
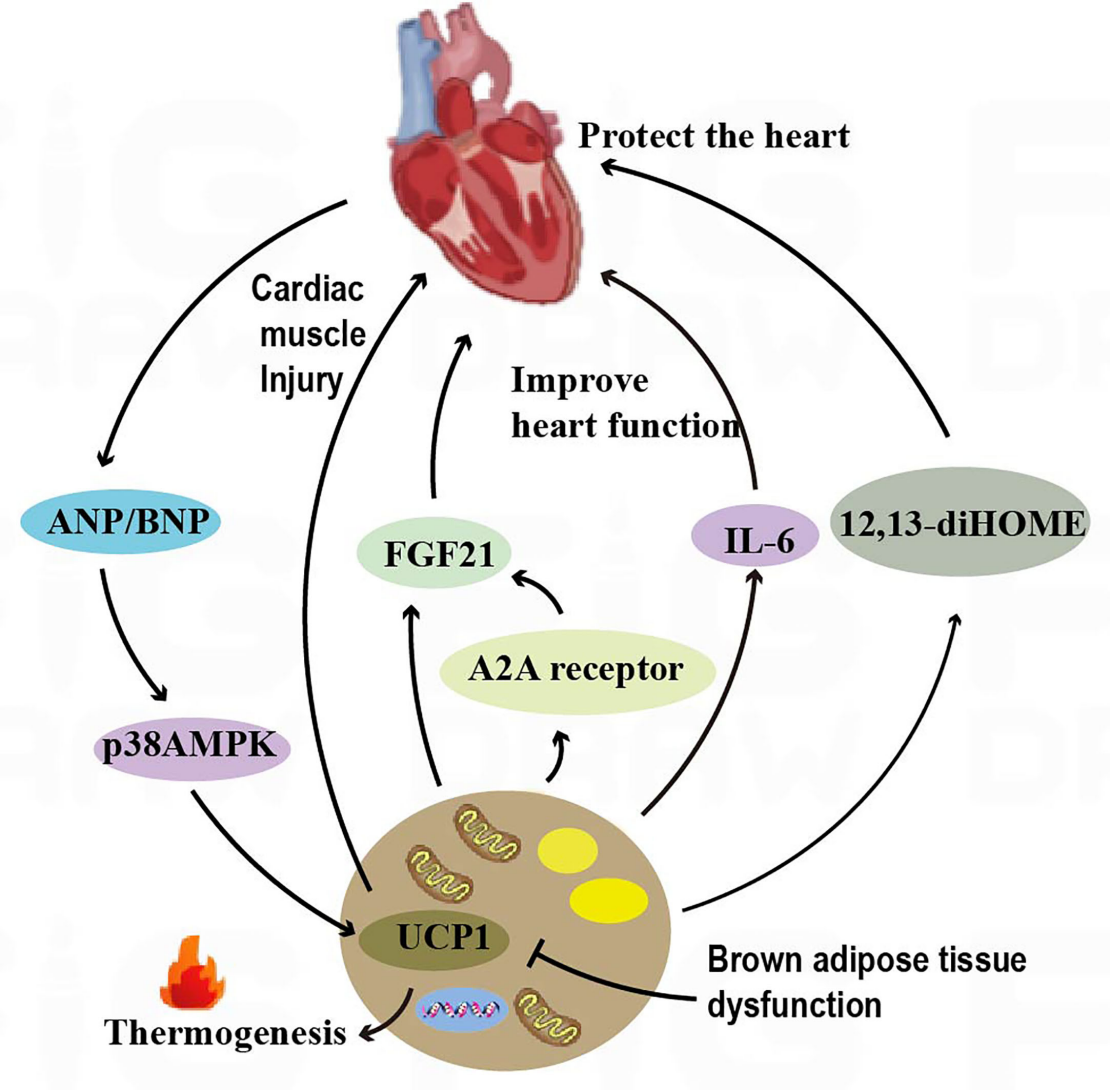
Crosstalk between adipose tissue and the heart. BAT regulates cardiac remodeling through the A2AR/FGF21 pathway by secreting cytokines. The IL-6 released by BAT increases cardiac matrix oxidation, protecting the heart from hypertrophy and oxidative stress. BAT also regulates cardiac function by releasing the fatty factors 12,13-diHOME. ANP and ventricular natriuretic peptide ANP increase the expression of UCP1 through the p38 MAPK pathway to mediate thermogenesis.

BAT is thought to have a role in improving heart function, and the presence of BAT is negatively associated with the incidence of heart disease (97). The dysfunction of brown adipose tissue refers to the inability of brown adipose tissue in the body to function properly, leading to a disorder in energy metabolism. The characteristic of BAT dysfunction associated with UCP1 deficiency is impaired thermogenic capacity. In a catecholamine-induced myocardial injury model in mice, UCP1 deficiency resulted in BAT dysfunction, increased myocardial cell injury and adverse left ventricular remodeling in mice, and decreased survival in a mouse model of catecholamine-induced cardiomyopathy. Transplantation of functional BAT into UCP1-deficient mice rescued myocardial injury as well as increased survival (98) (**Figure 3**).

Ectopic adipose tissue can also negatively impact cardiac function. Research studies have shown that epicardial adipose tissue (EAT) may have a potential impact on cardiovascular (CV) risk (99–102). Abnormal adipogenesis in the epicardium can lead to the secretion of pro-inflammatory adipokines, which can in turn lead to atrial and ventricular fibrosis (99). Thicker and dysfunctional epicardial adipose tissue (EAT) can promote the development and progression of coronary atherosclerosis. In addition, EAT may release mediators directly into the vessels of the coronary artery wall through a process known as “vascular secretion.” The pro-inflammatory and pro-fibrotic effects of dysfunctional EAT may ultimately impair cardiac structure and function (100).

#### 4.2.2 BAT secretes cytokines to regulate cardiac function

The adenosine A2A receptor is a type of G protein-coupled receptor that plays an important role in the cardiovascular system. In mice, knockdown of the adenosine A2A receptor leads to BAT dysfunction, which can in turn accelerate cardiac remodeling in hypertension. Brown adipose tissue is capable of secreting fibroblast growth factor 21 (FGF21)^6^, which can in turn influence cardiac function. Studies have shown that knockdown of FGF21 specifically in brown adipocytes can attenuate the modulatory effects of A2A receptor agonists on cardiac remodeling in hypertension. Conversely, activation of the A2A receptor in BAT can promote the release of FGF21 and improve cardiac function. This finding reveals an endocrine role of BAT in hypertensive cardiac remodeling through secretion of cytokines activating the A2AR/FGF21 pathway (8) (**Figure 3**). IL-6^7^ release from BAT increased cardiac matrix oxidation and protected the heart from hypertrophy and oxidative stress (10) (**Figure 3**). In addition, brown adipose tissue can modulate cardiac function by releasing adipokines such as 12,13-diHOME^8^. Studies have shown that overexpression of 12,13-diHOME can counteract the deleterious effects of a high-fat diet on cardiac function and remodeling. Furthermore, acute injection of 12,13-diHOME has been shown to increase cardiac hemodynamics through direct effects on cardiomyocytes (11) (**Figure 3**). Leptin secreted by adipose tissue is an adipokine with pleiotropic effects that are associated with cardioprotection. Leptin overexpression in mouse models was found to counteract inflammation in the heart and adipose tissue by regulating gene expression (103).

#### 4.2.3 Factors produced by the heart can regulate BAT activity

Natriuretic peptides, including atrial natriuretic peptide (ANP) and ventricular natriuretic peptide (BNP), can be produced by the heart (**Figure 3**). Both activate PPARγ coactivator-1α (PGC-1α)^9^ and UCP1 expression in human adipocytes, induce mitochondrial production, and increase uncoupling and total respiration. At low concentrations, ANP and β-adrenoceptor agonists were added to enhance the expression of brown adipose and mitochondrial markers in a p38 MAPK-dependent manner. Infusion of BNP into mice significantly increased the expression of UCP1 and PGC-1α in white adipose and brown adipose tissues and increased respiration and energy expenditure (25).

### 4.3 Crosstalk between adipose tissue and the liver

#### 4.3.1 Effect of adipose tissue and its secretory factors on liver function

**Figure 4:**
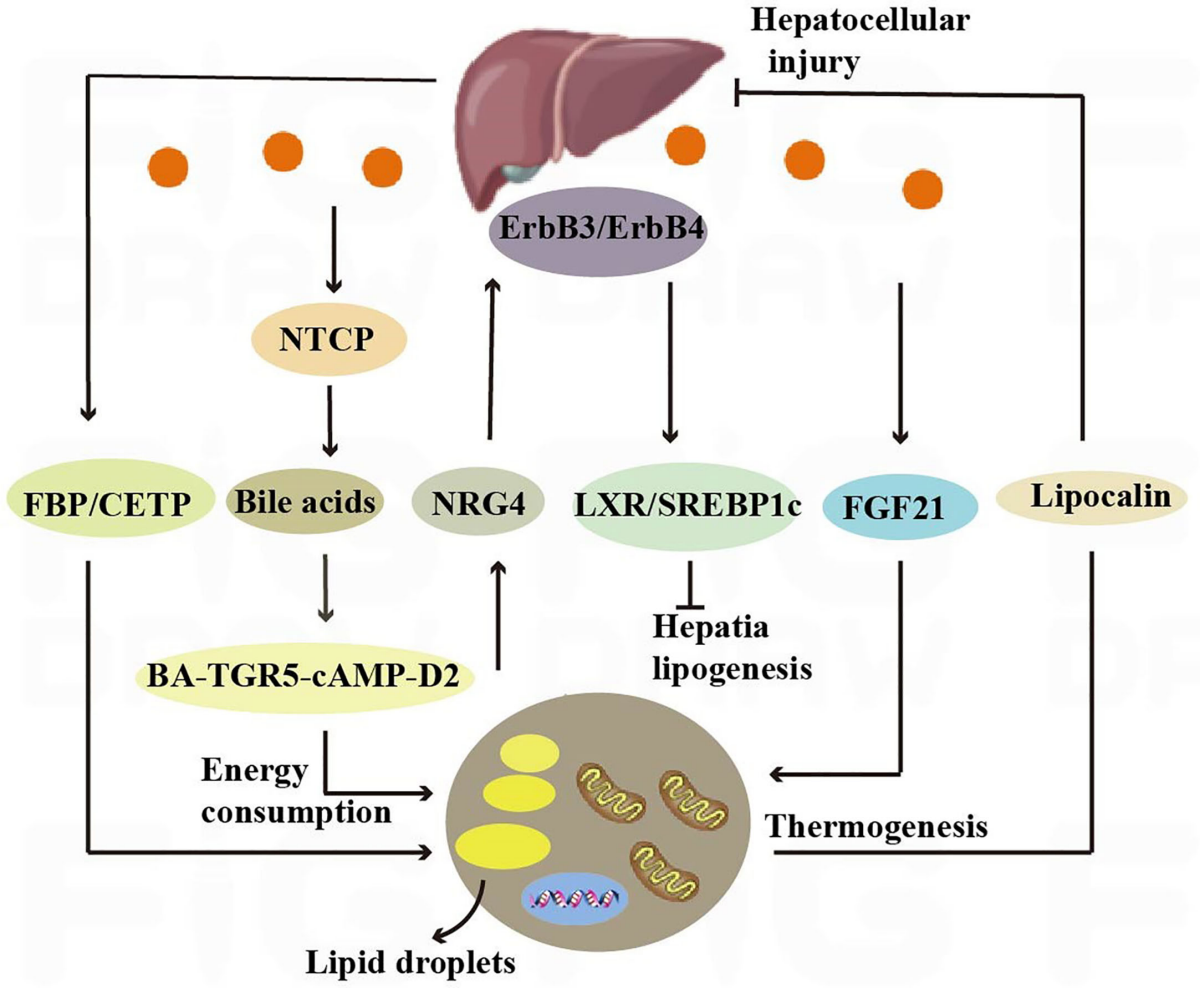
Crosstalk between adipose tissue and the liver. Nrg4 activates ErbB3/ErbB4 signaling in liver cells, regulating LXR/SREBP1c-mediated fat generation. Liver secretes bile acids that induce energy expenditure by promoting intracellular thyroid hormone activation. The expression of the sodium-taurocholate cotransporting polypeptide (NTCP) on the surface of liver cells increases plasma bile acid levels, alleviating liver steatosis and reducing plasma cholesterol levels. Hepatic fructose-1,6-bisphosphatase (FBP) and cholesterol ester transfer protein (CETP) synthesized in the liver regulate food intake and brown adipose tissue activity to alleviate obesity.

Neuromodulatory protein 4 (Nrg4) is highly expressed in brown adipose tissue, and it has been shown to reduce insulin resistance and hepatic steatosis by attenuating hepatic adipogenic signaling. Mechanistically, Nrg4 activates ErbB3/ErbB4 signaling in hepatocytes and regulates LXR/SREBP1c-mediated *de novo* adipogenesis in a cell-autonomous manner (17). A 2019 study showed that alcohol consumption stimulates hypothalamic neural circuits and sympathetic nerves innervating BAT and increases BAT uncoupling protein 1 expression and activity in a BAT sympathetic-dependent manner, thereby inhibiting lipid transport to the liver, suggesting that brown adipose tissue activation attenuates alcohol-induced hepatic steatosis and injury in mice. Brown adipocytes also secretes the adiponectin^10^ to inhibit hepatocyte injury and death (18). Leptin secreted by adipose tissue has a role in regulating diet and energy metabolism, and exogenous administration of leptin modulates the activity of water channel proteins thereby ameliorating nonalcoholic fatty liver disease (NAFLD) in mice, while leptin restores the coordinated action of Aquaporin-9(AQP9), thereby preventing excessive lipid accumulation in the liver in WAT and obesity (13) (**Figure 4**).

Adipose tissue serves as a major source of circulating exosomal miRNAs that are associated with improved glucose tolerance and hepatic FGF21 levels (104). FGF21 mediates a bidirectional communication axis between BAT and liver, and hepatic FGF21 contributes to thermogenic activation of brown fat in neonates (105). Patatin-like phospholipase domain-containing protein 3 (PNPLA3) is an adipocyte-specific nutrient-regulating transmembrane protein in mice called adiponectin (106), a hepatic lipase with triglyceride hydrolase activity. Replacement of isoleucine at protein position 148 by methionine can lead to loss of PNPLA3 function and hinder remodeling of monounsaturated and polyunsaturated fatty acid esters in favor of retention in the liver, which is an important factor contributing to the development of fatty liver (107) (**Figure 4**).

#### 4.3.2 Effect of liver on heat production in brown adipose tissue and browning of white fat

The liver, through metabolic conversion, can produce acylcarnitine to provide fuel for brown fat thermogenesis. White adipose tissue provides the liver with long-chain fatty acids, allowing the liver to produce plasma acylcarnitine as a fuel source for peripheral tissues in mice (108). The liver also contributes to energy consumption through other endocrine pathways, and it has been shown that hepatic secretion of bile acids induces energy expenditure by promoting intracellular thyroid hormone activation. The increased energy expenditure in brown adipose tissue observed after the administration of bile acids to mice can prevent obesity and insulin resistance, and it has been demonstrated that this effect is mediated by the BA-TGR5-cAMP-D2 signaling pathway (26) (**Figure 4**).

Hepatocyte surface expression of sodium-taurocholic acid cotransport protein (NTCP) reduces hepatic clearance of plasma bile acids, increases plasma bile acid levels, reduces diet-induced obesity, attenuates hepatic steatosis, and lowers plasma cholesterol levels. NTCP, G protein-coupled bile acid receptor (TGR5) double knockout mice are equally protected from diet-induced obesity as NTCP single knockout mice. NTCP deficiency was associated with increased uncoupled respiration in brown adipose tissue, leading to increased energy expenditure (109) (**Figure 4**). Bile acids are also involved in TGR5 signaling promoting mitochondrial division and beige remodeling in white adipose tissue (27).

Certain enzymes present in the liver also play an active role in thermogenesis and weight maintenance. Hepatic fructose-1,6-bisphosphatase (FBP) is a regulatory enzyme in gluconeogenesis, and an increase in FBP reduces food intake by 15% without reducing energy expenditure. The decrease in food consumption was associated with increased levels of leptin and fatty acid oxidation, decreased appetite stimulating neuropeptide, neuropeptide Y (110). Cholesteryl ester transfer protein (CETP), which is synthesized by the liver, can also reduce obesity by enhancing lipolysis and brown adipose tissue activity (28). All of the above studies have shown that crosstalk between the liver and adipose tissue is necessary to maintain liver function and systemic energy homeostasis (**Figure 4**).

### 4.4 Crosstalk between adipose tissue and skeletal muscle

#### 4.4.1 Exercise increases adipose tissue browning by inducing the production of multiple factors in the circulation

**Figure 5:**
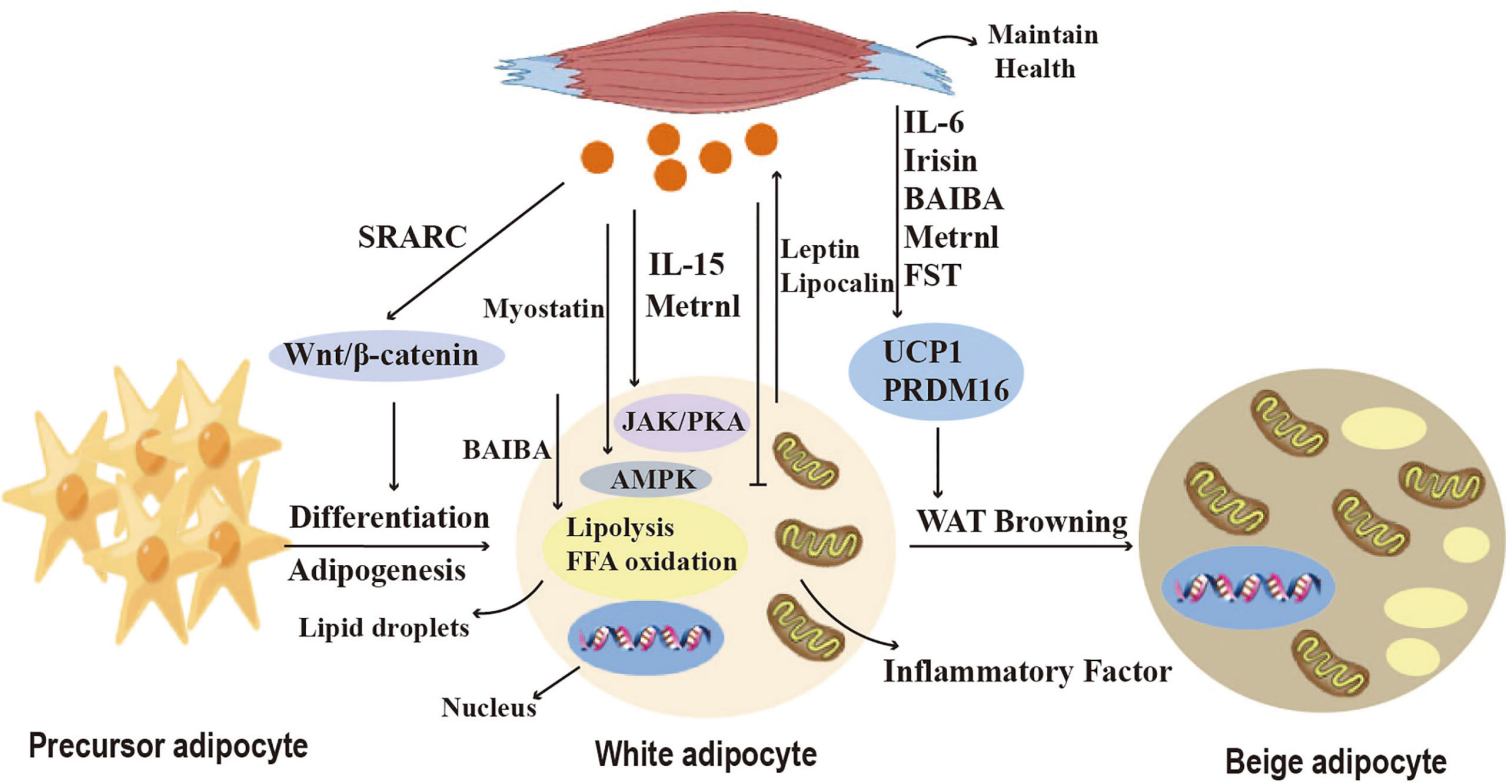
Crosstalk between adipose tissue and skeletal muscle. Myokines regulate adipogenesis, lipid metabolism, adipokine secretion and the browning of WAT. Myostatin and IL-15 affect lipid breakdown through the JAK, PKA, and AMPK pathways. SPARC and Irisin regulate pre-adipocyte differentiation and lipid synthesis *via* the Wnt/b-catenin pathway, and BAIBA induces lipolysis by increasing mitochondrial FFA oxidation in adipocytes. Metrnl inhibits the secretion of pro-inflammatory cytokines. Irisin, FST, IL-6, BAIBA, and Metrnl can induce browning of white adipose tissue (WAT) and affect thermogenesis.

Myokines are a collection of cytokines and other small proteins released by skeletal muscles. Skeletal muscles can synthesize and secrete various myokines (111) (**Figure 5**). It has been shown that exercise promotes BAT activation, as well as white adipose tissue deposition to the subcutis and WAT browning (29) (**Figure 5**). Exercise induces the secretion of myokines to mediate crosstalk between adipose tissue and skeletal muscle. Studies have shown that exercise induces IL-6 secretion in skeletal muscle, which regulates WAT and skeletal muscle metabolism and helps maintain metabolic homeostasis during exercise recovery (30). It has been shown that IL-6 injection can increase iWAT UCP1 mRNA content in WT mice (31). Exercise can also induce thermogenesis by inducing the production of other myokines, irisin, a hormone-like myokine that is produced in large amounts in skeletal muscle during exercise, acts on white adipocytes to induce a browning response and subsequently activates non-shivering thermogenesis (32). β-aminoisobutyric acid (BAIBA) is a myokine that increases in plasma concentrations after exercise, and promotes the activation of brown adipose tissue, as well as the browning of white adipose tissue. BAIBA also induces lipolysis by increasing FFA oxidation in adipocytes’ mitochondria (33).

Nickel riboflavin (METRNL) is a circulating factor induced in muscle after exercise and in adipose tissue after cold exposure. increased METRNL at circulating levels stimulates energy expenditure, improves glucose tolerance, and is associated with expression of genes related to thermogenic and anti-inflammatory cytokines in beige fat. Its mechanism of action is to stimulate increased IL-4 expression and promote selective activation of adipose tissue macrophages, leading to increased thermogenic and anti-inflammatory gene expression (34). Brain-derived neurotrophic factor (BDNF) is a kind of neurotrophic factor that is widely distributed in multiple regions of the central nervous system (CNS), including the cerebral cortex and hippocampus. A study has discussed the impact of aerobic exercise on BDNF. In the experiment, rats were engaged in aerobic exercise, and it was found that aerobic exercise could increase the levels of BDNF. This indicates a close relationship between BDNF and exercise (112). Stimulating the hypothalamic expression of BDNF through environmental stimuli can induce browning of white adipocytes and increase energy expenditure, but its mechanism is not yet clear (113). Follistatin (FST), as a myocyte factor, can promote the secretion of Irisin in subcutaneous fat through the AMPK-PGC1α-Irisin signaling pathway, inducing WAT browning (35). Myocyte factors can also regulate the lipid metabolism of adipocytes. Studies have shown that IL-15 is lowly expressed in pig adipose tissue and that interleukin-15 (IL-15) regulates lipid accumulation by enhancing lipolysis or inhibiting lipid synthesis through JAK and PKA pathways (36). SPARC, also known as osteonectin or BM-40, is a matricellular protein that belongs to the family of extracellular matrix proteins. It is a cysteine-rich acidic protein and sometimes referred to as an osteogenic protein. SPARC is present in the secretome of various cells, including muscle and bone cells, and is predominantly expressed in subcutaneous adipose tissue. SPARC negatively regulates adipocyte differentiation and adipogenesis in white adipose tissue (WAT) through activation of the Wnt/β-catenin pathway (14). These findings indicate the important role of various myocyte factors in regulating adipocyte browning, thermogenesis, and lipid metabolism.

#### 4.4.2 Effect of secretory factors of adipose tissue on skeletal muscle and exercise capacity

WAT adipose tissue can secrete a variety of adipokines, such as adiponectin and leptin (19) (**Figure 5**). Adiponectin activates AMPK in skeletal muscle and stimulates fatty acid oxidation by activating p38 mitogen-activated kinase and PPARa (20). Adiponectin also significantly reduces ceramide levels in exercise-oxidized muscle (15). Leptin promotes skeletal muscle lipid metabolism and fatty acid utilization, as well as energy production to reduce skeletal muscle lipid accumulation (16). Additionally, leptin increases muscle mass by inducing myocyte proliferation factors and negative regulators that inhibit muscle growth, as well as atrophy markers MAFbx and MuRF1 (21). In addition, other adipokines such as retinol-binding protein 4 and endolipin play important roles in regulating the regulation of glucose homeostasis in skeletal muscle as well as insulin uptake (22).

Brown adipose tissue also secretes adipokines, called brown adipokines (BATokines). Overexpression of IRF4 in brown adipocytes decreases serum muscle growth inhibitory hormone and increases muscle motility (12). Thus, IRF4 and muscle growth inhibitor mediate the crosstalk between brown adipose tissue and skeletal muscle. Exercise induces BAT production of 12,13diHOME, which increases skeletal muscle fatty acid uptake and oxidation, and acute 12,13-DHOME treatment in mice exerts the same effect (9). Fibroblast growth factor 21 (FGF21), released by BAT, increases thermogenesis by inducing an increase in BAT and mitochondrial cristae in skeletal muscle under cold exposure conditions (114). In addition, adipocyte-derived exosomes can enter skeletal muscle cells, and exosomal miR-27a induces insulin signaling by inhibiting PPARγ-induced insulin resistance in skeletal muscle (115). Exosomal miR-130b downregulates PGC-1a to reduce mitochondrial oxidative capacity (37). All the above studies have shown that crosstalk between adipose tissue and skeletal muscle is necessary to maintain energy homeostasis (**Figure 5**).

### 4.5 Crosstalk between adipose tissue and gut

#### 4.5.1 Gut-brain-BAT crosstalk induces BAT thermogenesis

**Figure 6:**
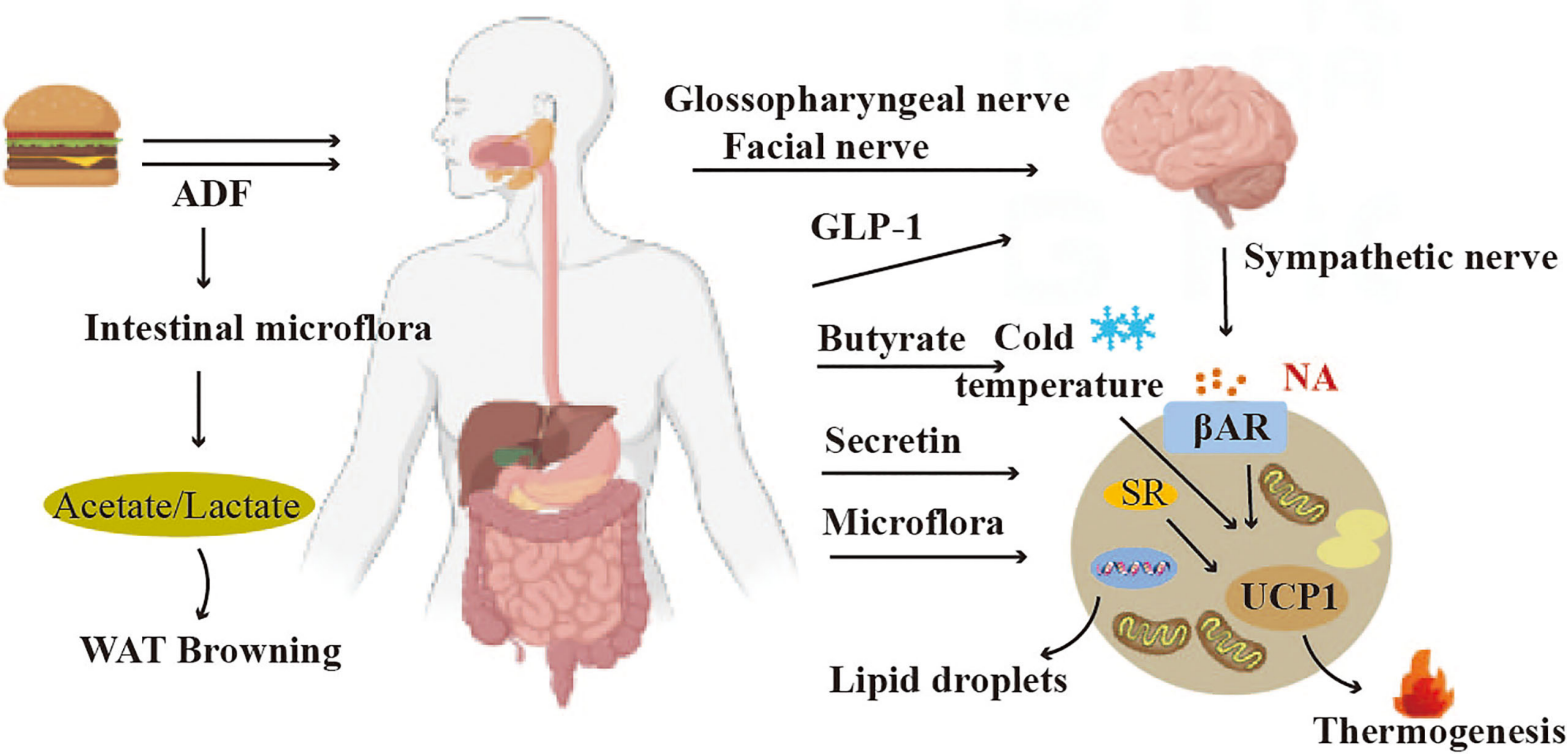
Crosstalk between adipose tissue and gut. The hormones GLP-1 secreted by the gastrointestinal tract can trigger Gut-brain-BAT crosstalk to induce BAT thermogenesis. The gut microbiota can regulate the functions of both BAT and WAT.

The gut-brain-BAT axis is a network of information exchange connecting the gut to the brain and to adipose tissue. Studies have shown that factors secreted by the gastrointestinal tract can trigger gut-brain-BAT crosstalk to induce BAT thermogenesis. Glucagon-like peptide 1 (GLP-1) is a peptide hormone secreted by intestinal L cells, and its receptor agonist (GLP-1R) is a new antidiabetic agent in recent years.GLP-1 can regulate white adipose tissue and brown adipose tissue thermogenesis by activating the sympathetic nervous system (38, 116) (**Figure 6**). The gut hormone glucagon, when released during feeding, triggers gut-brain-brown adipose tissue (BAT) crosstalk to mediate meal thermogenesis, which leads to satiety. Mechanistically, the meal-related elevation of circulating glucagon activates BAT thermogenesis by binding to glucagon receptors in brown adipocytes and promoting lipolysis. This, in turn, sends signals to the brain to produce satiety (117) (**Figure 6**). The brown fat cells in turn send signals back to the brain to produce a feeling of satiety.

#### 4.5.2 Gut microbiota regulates BAT and WAT functions

Gut microbiota is closely associated with brown adipose tissue thermogenesis, and white adipose tissue thermogenesis (118) (**Figure 6**). Studies have shown that alternate-day fasting stimulates white adipose tissue browning and improves obesity and insulin resistance. The mechanism involves a shift in the composition of the intestinal microbiota, resulting in elevated fermentation products acetate and lactate, and upregulation of monocarboxylate transporter protein 1 expression in beige adipocytes (119). The gut microbiota is also able to regulate lipid metabolism in BAT. Deficiency of gut microbiota stimulates hepatic and BAT lipolysis, while inhibiting lipogenesis (120). By transplanting gut microbiota into cold-induced mice, the function of brown adipose tissue in mice could be improved (121). However, In 2019, a study found that depleting the microbiota of mice through antibiotic treatment or asepsis decreased the expression of uncoupling protein 1 (UCP1) and inhibited the browning process of white adipose tissue, resulting in impaired thermogenic capacity of brown adipose tissue. The study found that feeding mice with butyrate, a bacterial metabolite, increased their thermogenic capacity. These findings suggest that the gut microbiota may play a role in upregulating thermogenesis in cold environments, potentially through the action of butyrate (122). The intestinal microorganism E. faecalis and its metabolite myristic acid (MA) have been shown to reduce obesity by activating brown adipose tissue and promoting beige fat formation (123).

## 5 Mechanisms of the role of adipose tissue in disease onset and progression and in disease treatment

### 5.1 Mechanism of the role of adipose tissue in disease onset and progression

#### 5.1.1 The mechanisms of adipose tissue in the occurrence and development of metabolic diseases

Adipose tissue dysfunction is associated with the development of several diseases, and understanding the molecular and cellular mechanisms by which adipose tissue contributes to these diseases will facilitate the development of adipose tissue-based treatments. Adipose tissue has a primary function as a nutrient buffer, protecting other tissues from nutrient toxicity, and in obese patients, the nutrient buffering capacity of adipose tissue fails, leading to systemic toxicity (54). Mutations in genes associated with fat storage and lipolysis can likewise lead to metabolic diseases (124). Obesity leads to adipose tissue overnutrition, and hypertrophy is an adaptive mechanism used by adipose tissue to buffer overnutrition, but hypertrophic adipose tissue over 100 μm in diameter leads to a state of cellular hypoxia, which triggers a cellular stress response, which leads to an inflammatory response from adipocyte apoptosis. Studies have shown that diabetic patients have increased adipocyte hypertrophy in their adipose tissue compared to non-diabetic patients. This indicates a correlation between obesity and diabetes, and further highlights diabetes as a contributing factor in metabolic diseases. Adipose tissue fibrosis restricts adipocyte hypertrophy and has beneficial effects on systemic metabolism. This finding suggests that adipose tissue fibrosis could be a potential target for manipulating adipocyte metabolism (125). When adipocytes reach their hypertrophic limit, lipids spill over into the systemic circulation, leading to tissue toxicity. When visceral adipose tissue reaches its nutrient storage capacity, free fatty acids flood into the liver through the portal system, leading to hepatic steatosis, which progresses to steatohepatitis (126).

#### 5.1.2 The mechanisms of adipose tissue distribution heterogeneity in the occurrence and development of diseases

The distribution of adipose tissue is heterogeneous and can be divided into subcutaneous and visceral areas. An increased proportion of visceral adipose tissue is associated with an increased risk of metabolic diseases. As we age, subcutaneous adipose tissue shifts to the viscera. The trend of obesity in women is associated with increased expression of lipoprotein lipase in subcutaneous adipose tissue (127). Studies have shown that Fyn knockout mice on normal and high-fat diets have increased glucose clearance and systemic insulin sensitivity, increased fatty acid utilization and energy expenditure resulting in reduced obesity. Analysis shows that Fyn knockout mice shift adipose tissue to the subcutis rather than the viscera, which reduces adipose tissue inflammation associated with T cell and macrophage infiltration (128). The redistribution of adipose tissue by Fyn would serve as a new therapeutic strategy.

#### 5.1.3 The mechanisms of adipose tissue in the occurrence and development of cardiovascular diseases

The dysfunction of adipose tissue mainly manifests as the disturbance of its metabolic function and the abnormal secretion of hormones. Specifically, it can manifest as dyslipidemia, enhanced inflammatory response, abnormal hormone secretion, and activation of the renin-angiotensin-aldosterone system (RAAS). Adipose tissue dysfunction is closely related to the development of cardiovascular diseases. In obese patients, systemic or local inflammation and insulin resistance can cause macrophages in adipose tissue to switch from anti-inflammatory and anti-atherosclerotic to pro-inflammatory and pro-atherosclerotic. Adipose tissue expansion and dysfunction contribute to the development of cardiovascular disease through direct and indirect mechanisms. It is known that obesity may induce hypertension through visceral adipose tissue expansion causing mechanical compression of the kidney, sympathetic nervous system activity and activation of the renin-angiotensin-aldosterone system (RAAS) (56).

### 5.2 Mechanism of the role of adipose tissue in disease treatment

Thermogenic adipose tissue is known to combat obesity and metabolic disorders, and increasing the amount and activity of brown adipose provides new strategies for the treatment of diseases such as obesity. Studies have shown that there is therapeutic potential of activating thermogenic adipose tissue or converting white adipocytes into thermogenic adipocytes using pharmacological and genetic approaches. Myrbetriq is a β3 adrenergic stimulant that acts on β3 adrenergic receptors in bladder smooth muscle. Some studies have also suggested that Myrbetriq has a stimulatory effect on human brown adipose tissue thermogenesis, which may be a promising pharmacological therapeutic approach for metabolic disorders (129). Capsaicin also has anti-obesity potential. By ingesting capsaicin, it can improve energy expenditure and fat oxidation in obese patients (130). A 2016 study showed that a combination of mild cold exposure and capsaicin synergistically promoted beige adipocyte development and improved obesity through the beta2-adrenoceptor signaling pathway (131). GLP1 agonists can activate BAT thermogenesis through the mouse brain, but it has the opposite effect in humans (132).

Gene therapy allows for the selective introduction of target genes into target cells or correction of target gene expression for disease treatment. UCP1 is known to mediate brown adipose tissue thermogenesis, and increased UCP1 would facilitate mitochondrial uncoupling. Introduction of PRDM16 gene and C/EBP-β into human dermal fibroblasts induced brown adipose tissue production and significantly reduced diet-induced obesity and insulin resistance in a UCP1-dependent manner after transplantation. Thus, brown adipose tissue can be generated by cellular reprogramming, suggesting that gene therapy is a promising therapeutic strategy for metabolic disorders (133). Studies have shown that human thermogenic adipocytes specifically express long non-coding RNALINC00473, an RNA that is highly correlated with UCP1 expression, demonstrating that LINC00473 is a key regulator of human thermogenic adipocyte function, providing an alternative strategy for gene-based brown adipose tissue activation (134). The application of CRISPR-Cas9-mediated homologous recombination-independent approaches efficiently inserted recombinant UCP1 into the endogenous UCP1 locus in pigs, and pigs with recombinant UCP1 exhibited better cold tolerance (135). In conclusion, gene therapy in human adipocytes or stem cells is a potential therapeutic approach.

The use of pharmacological treatment to induce thermal differentiation of stem cells is one type of cell therapy. In one study, a specific thrombopoietin mixture was used to differentiate human pluripotent stem cells into functional classical brown adipocytes without exogenous gene transfer. The resulting human pluripotent stem cell-derived brown adipose tissue exhibited β-adrenergic receptor-stimulated activation of respiration and thermogenesis, as well as enhanced glucose tolerance (136). Human precursor white adipocytes were genetically engineered using CRISPR -Cas9 to activate endogenous UCP1 expression, resulting in the creation of human brown-like (HUMBLE) cells. Obese mice that received HUMBLE cell transplants showed improved glucose tolerance and insulin sensitivity, as well as increased energy expenditure. These findings demonstrate the potential of using CRISPR-Cas9 technology to modify human white adipocytes and induce a brown adipose-like phenotype, offering opportunities for cell-based therapies to combat obesity and diabetes (137). Overall, these studies highlight the potential of adipocyte-based therapeutic approaches for addressing obesity and metabolic diseases.

## 6 Conclusion

Brown adipocytes and white adipocytes play different roles in heat production and energy storage. Adipose tissue not only serves as a site for energy storage and release but also a complex ecological niche that influences the activity of stem cells and immune cells as well as the function of other organs, which is essential in maintaining its function and internal environment stability. Adipose tissue controls systemic metabolism through remote interactions with other organs, and growing evidence shows the impact of inter-organ communication between adipose tissue and other organs on physiological and pathological processes. Brown adipose tissue (BAT) has great potential in combating obesity and metabolic disorders and increasing its quantity or activity through genetic, pharmacological, and cellular therapeutic methods to prevent and treat obesity, type 2 diabetes, and related metabolic disorders.

However, while various therapeutic strategies for brown and beige adipocytes have shown promising results in rodent models, they cannot be simply applied to humans. A 2020 study suggested that human BAT thermogenesis is not mediated by β3-adrenoceptors (β3-AR) but rather by β2-adrenoceptors, which is in contrast to rodents (138). Other studies imply that β1 adrenoceptor may be the main regulatory factor for human brown adipocyte thermogenesis (139). Consequently, differences in metabolic features between brown adipocytes from mice and humans warrant further investigation. Furthermore, while the aim of obesity treatment methods is to increase the quantity of thermogenic adipose tissue, approaches to tackle disease related to heterogeneity in adipose tissue distribution, and functional disturbances of adipose tissue on cardiovascular disease treatment still require further research. In conclusion, ongoing advances in fundamental knowledge and innovative technologies hold unlimited hope for developing therapies for adipose tissue dysfunction-related diseases.

## Author contributions

SW and YL drafted the manuscript. JC and YH prepared the graphs. XS, XL and WM edited the manuscript. All authors contributed to the article and approved the submitted version.

## Footnotes

1 PRDM16 is formed by the combination of PRDM family members PRDM3 and PRDM5, and its encoded protein is involved in the regulation of many cellular processes, including differentiation, proliferation, and apoptosis.

2 BMP8b is a signaling protein, also known as a growth factor, belonging to the bone morphogenetic protein superfamily. It plays an important role in bone formation and repair as well as fat metabolism.

3 NRG4 is a signaling protein secreted by adipose tissue, also known as a adipokine, and belongs to the neuregulin family. It plays an important role in regulating energy metabolism, fat distribution, and insulin sensitivity.

4 CXCL14 is a chemokine, a protein that promotes the movement of white blood cells, which mainly exists in various tissues and cells such as the gastrointestinal tract, muscles, and adipose tissue, and plays an important role in immune regulation, tumor growth, angiogenesis, and nerve development.

5 Leptin is a hormone secreted by fat cells and considered a key regulator of body weight and energy metabolism.

6 FGF21 is a protein secreted by the liver and other tissues, belonging to the fibroblast growth factor (FGF) family, and plays a role in regulating glucose and lipid metabolism.

7 IL-6 is a cytokine interleukin-6 that is a pro-inflammatory protein. IL-6 is produced by various cells, including T cells, B cells, liver, and monocytes.

8 12,13-diHOME is a metabolite of fatty acid metabolism in the blood, which is formed by the action of the fatty acid hydroxylase in adipose tissue on fatty acids.

9 PGC-1α is an important transcriptional coactivator that can promote mitochondrial biogenesis and synthesis. It is widely expressed in tissues including liver, heart, muscle and adipose.

10 Adiponectin is a protein hormone secreted by adipocytes and is one of the representatives of healthy adipocytes. It plays an important physiological role in glucose and lipid metabolism, energy metabolism, inflammatory processes, and other areas.
